# Selection of an Optimal Abrasion Wheel Type for Nano-Coating Wear Studies under Wet or Dry Abrasion Conditions

**DOI:** 10.3390/nano10081445

**Published:** 2020-07-24

**Authors:** Li-Piin Sung, Yu-Fan Chung, David G. Goodwin, Elijah J. Petersen, Hsiang-Chun Hsueh, Paul Stutzman, Tinh Nguyen, Treye Thomas

**Affiliations:** 1 Engineering Laboratory, National Institute of Standards and Technology (NIST), Gaithersburg, MD 20899, USA; moonlist31@gmail.com (Y.-F.C.); david.goodwin@nist.gov (D.G.G.J.); d9631837@oz.nthu.edu.tw (H.-C.H.); paul.stutzman@nist.gov (P.S.); tnguyen6718@outlook.com (T.N.); 2 Materials Measurement Laboratory, NIST, Gaithersburg, MD 20899, USA; 3 Office of Hazard Identification and Reduction, U.S. Consumer Product Safety Commission, Bethesda, MD 20814, USA; tthomas@cpsc.gov

**Keywords:** nanorelease, nanofiller, nanotechnology environmental health and safety, laser scanning confocal microscopy, abrasion, wheel, wear, nanocoating

## Abstract

Nanocoatings have numerous potential applications in the indoor environment, such as flooring finishes with increased scratch- and wear-resistance. However, given concerns about the potential environmental and human health effects of nanomaterials, it is necessary to develop standardized methods to quantify nanomaterial release during use of these products. One key choice for mechanical wear studies is the abrasion wheel. Potential limitations of different wheels include the release of fragments from the wheel during abrasion, wearing of the wheel from the abrasion process, or not releasing a sufficient number of particles for accurate quantitative analysis. In this study, we evaluated five different wheels, including a typically used silicon oxide-based commercial wheel and four wheels fabricated at the National Institute of Standards and Technology (NIST), for their application in nanocoating abrasion studies. A rapid, nondestructive laser scanning confocal microscopy method was developed and used to identify released particles on the abraded surfaces. NIST fabricated a high performing wheel: a noncorrosive, stainless-steel abrasion wheel containing a deep cross-patch. This wheel worked well under both wet and dry conditions, did not corrode in aqueous media, did not release particles from itself, and yielded higher numbers of released particles. These results can be used to help develop a standardized protocol for surface release of particles from nanoenabled products using a commercial rotary Taber abraser.

## 1. Introduction

One recent innovation to substantially improve the properties of polymeric materials has been to incorporate nanoparticles (i.e., particles having at least one external dimension less than 100 nm) as nanofillers [1,2,3]. Although the use of nanofillers has resulted in a projected multibillion dollar industry in coatings, paints, and adhesives [4], these products could be exposed to mechanical stresses (e.g., abrasion) throughout their lifecycle, to weathering (e.g., UV radiation) while exposed to outdoor environments [5,6,7], and to additional stresses, such as incineration and chemical dissolution during disposal and recycling [6,7,8,9], that have the potential to cause nanoparticle release. Although all coatings, paints, and adhesives will be subjected to these stresses, they are especially important for products with nanofillers, given concerns about potential environmental and human health impacts from released particles [10,11,12,13,14,15,16,17,18,19,20,21].

These potential concerns make the development of quantitative methods for determining the release of nanoparticles after various stresses a key research topic. Substantial research on release after drilling, polishing, sanding, and abrasion [8,22,23,24,25,26,27,28,29] of nanoparticle-containing products has revealed that nanoparticles are typically not released by themselves, but they are more often at least partly embedded in released fragments of the material matrix [6]. Although there have been numerous studies on nanoparticle release during outdoor applications, where weathering or a combination of weathering and abrasion may cause release [5,30,31,32,33,34,35,36,37,38,39,40,41], few studies have been conducted for release in the indoor environment, where people have the potential for exposure to nanoparticles applied as additives in flooring coatings and interior paints. Nanoparticle release can occur in the indoor environment as a result of scraping, mopping, and other abrasive activities. An additional complication related to assessing the release of nanoparticles that accumulate on a material surface after abrasion is that methods are not yet developed, unlike the fairly mature techniques used for quantifying particles released into air [22,23,24,25,26,28,30].

One challenge in previous abrasion studies is that some wheels were shown to release particles themselves during the abrasion process [30]. In addition, common commercial abrasive wheels, which are composed of alumina or silica particles embedded in a rubber binder, are not suitable for studies of nanoparticle release from nanocoatings where the nanoparticles (alumina or silica nanoparticles) in the nanofiller may be composed of the same elements. The alternative approach to avoid this issue is to use metallic wheels. However, the only commercially available metal wheel is composed of wear-resistant tungsten carbide. This wheel has a deep-cut and rough surface texture, which tends to hold released particles in the deep groves and may limit the number of released particles. Thus, research is needed to investigate the use of metallic wheels that have surface textures similar to those of the rubber binder wheels for generating sufficient released particles while avoiding release of material from the wheel itself. A systematic study of abrasion wheels is also needed since nanoparticles may harden coatings and affect the wear ability of coatings by abrasive wheels.

The main objective of this study was to identify an abrasion wheel or wheels that have an appropriate surface profile (i.e., surface roughness and pattern) to effectively abrade nanoparticle-containing polymer coatings and paints, without generating particles from the wheel itself during either dry or wet abrasion. Such a wheel(s) could become a “standard” or a recommended wheel for studies of nanoparticle release by the mechanical forces applied to coated and painted surfaces when assessing airborne release or released particles that remain on a sample surface. Previously, only a limited number of studies assessing airborne release of particles after mechanical wear of nanoenabled products have assessed the potential for the wheel itself to release particles [24,26,27]. This study tested five different wheels to evaluate their performance during abrasion experiments and the number of released particles on the sample surface after abrasion. Assessing the released particles on a sample surface is important because particles that remain on a sample surface after being released from a coating could lead to dermal or oral exposure. A secondary objective was the development of a laser scanning confocal microscopy (LSCM) method which can be used to quantify the concentration of surface-associated particles after the abrasion process even for rough surfaces which challenge analysis using other techniques such as scanning electron microscopy (SEM) and atomic force microscopy (AFM).

## 2. Materials and Methods 

### 2.1. Preparation of Materials for Abrasion

A commercial water-based polyurethane (PU) coating was chosen for this study and was applied to a wood substrate (Figure S1). Information about this polymeric material containing nanoparticles was provided to the National Institute of Standards and Technology (NIST) from a nanoparticle supplier (BYK USA Inc., Wallingford, CT, USA). The PU was a typical clear polymer coating used for hardwood flooring. The coating was purchased at a local home improvement store. There are no inorganic compounds indicated in the material safety data sheet (MSDS) for the PU coating issued by the manufacturer. However, according to the nanoparticle manufacturer, this PU coating contained small amounts of alumina (Al_2_O_3_) nanoparticles. Hereafter, the floor coating containing nanoparticles is designated as a nanocoating (NC).

Nanocoated wood disc specimens (9.6 cm in diameter) were prepared for wet and dry abrasion; detailed information is described in the Supplementary Material (SM). The dry film thicknesses of the NC were (256 ± 16) µm measured by a caliper (the number after the ± symbol indicates one standard deviation based on four measurements of independent samples). The coating material in the middle holes of the coated discs was removed by a drill before use. In addition to NC specimens on wood substrates for abrasion studies, free-standing films including dog bone samples, having a thickness of approximately 200 µm, were also prepared for evaluating a variety of properties of the NC materials. To produce a free-standing film, a single layer of NC was applied on a Mylar sheet, and the samples were then cured for two weeks.

A 1% nanoAl_2_O_3_ polyurethane floor coating and a 1.2% nanoTiO_2_ latex paint were also prepared to evaluate the potential for the rubber wheels to release particles themselves during abrasion. A full description of the sample preparation for these samples is provided in the SM. The 1% nanoAl_2_O_3_ sample was also evaluated to assess if there would be a different concentration of surface-associated particles in the absence and presence of the abrasion system vacuum during abrasion. All other measurements were performed on the NC described above.

### 2.2. Characterization of Initial Nanocoating Properties

Various initial properties of the NC that are relevant to this study were measured and are given in Table S1. Results are provided in the Supplementary Material (SM) for the following measurements of the NC using a free-standing film: mechanical properties of dry and water-immersed NC (Figure S2), thermogravimetric analysis (Figure S3), SEM and energy dispersive X-ray spectroscopy (EDS) (Figures S4 and S5), AFM (Figure S6), and LSCM (Figure S7). 

Mechanical properties (tensile strength and elastic modulus (E)) of the NC coating were measured using an Instron testing machine (Norwood, MA, USA) at a rate of 10 mm/min until break using dog bone specimens cut from free-standing films, according to the American Society for Testing and Materials (ASTM) standard D638. The tensile tests were performed on films after curing and immersion of the films in water for 1 d. These experiments were carried out to determine the effects of immersion in water on the modulus. Reported tensile strength and modulus values are the average of four specimens.

Glass transition temperatures (T_g_) (transition temperature from glassy state to rubbery state) of the NC were obtained using a dynamic mechanical analyzer (DMTA) (RSA III TA instruments, New Castle, DE, USA). Measurements were performed from −100 °C to 150 °C using a temperature ramp of 3 °C/min, with a frequency of 1.0 Hz and a strain of 0.5%. T_g_ was determined from the maximum of the tan δ peak and was the average of four measurements from four replicate specimens.

The quantity of inorganic material in the nanocoating (NC) was measured using a thermogravimetric analyzer (TGA) (TA instrument, New Castle, DE, USA). The analysis was carried out in air at a heating rate of 10 °C/min and a sample size of approximately 7 mg. The results were the average of five replicate samples.

Surface morphology of the initial NC surfaces was characterized by LSCM (Zeiss model LSM510, Carl Zeiss Microscopy, LLC., Thornwood, NY, USA) in reflection mode with a laser wavelength of 543 nm. LSCM images presented in this study are two-dimensional (2D) projections in the X-Y plane, obtained using an air lens (Objective magnification/Numerical aperture of 50×/0.5 or 150×/0.95) method. A typical 2D LSCM image (512 pixel × 512 pixel) was formed by summing the stack of images over the Z direction of the film. Pixel intensity represents the total amount of back-scattered light.

Atomic force microscopy was also used to characterize surface morphology of NC before abrasion. AFM measurements were carried out at ambient conditions (24 °C, 50% relative humidity) using a Dimension 3100 system (Bruker Dimension Icon AFM, Billerica, MA, USA) and silicon probes (TESP 70, Bruker Nano Inc., Camarillo, CA, USA). Both topographic (height) and phase images were obtained simultaneously using a resonance frequency of approximately 300 kHz for the probe oscillation and a free-oscillation amplitude of (62 ± 2) nm.

Scanning electron microscopy was performed using a Focused Ion Beam Scanning Electron Microscope (FIB SEM) FEI Helios NanoLab 650 (ThermoFisher Scientific, Hillsboro, OR, USA). Energy dispersive X-ray spectroscopy (EDXS) was performed using an Oxford Instruments X-Max 80 mm^2^ SDD-EDXS Detector (Oxford Instruments, NanoAnalysis, Concord, MA, USA). Additional SEM imaging and EDS analysis for a broader image area were performed using a JEOL JSM-7600F SEM (JEOL USA, Inc., Peabody, MA, USA).

Atomic force microscopy and SEM were only conducted on the free film samples prior to abrasion because the surface roughness values were too high after abrasion. Instead, LSCM was used to evaluate these samples. Similar LSCM results were obtained for NC wood disc samples and free films.

### 2.3. Instrumentation and Abrasion Parameters 

Abrasion of NC samples under dry and wet conditions was performed using a dual specimen table Taber rotary abraser (Model 5155, Taber, North Tonawanda, NY, USA). This abraser is widely used to evaluate the abrasion and wear resistance of coatings and paints and is specified in several international standards, including ASTM D 4060-14 and ISO 5470-1: 2016 (en). This type of instrument has also been used by researchers to study nanoparticle release from polymer nanocomposites due to mechanical forces [24,26,27]. The force exerted by the abrasion test simulates mechanical forces applied to organic coatings and paints from activities, such as walking, chair movement, polishing actions, and rubbing. The abraser consists of two vertical abrasive wheels rotating about a horizontal axis that abrades the material continuously while the horizontal specimen is rotating about a vertical axis at a fixed speed. The abrasion/rubbing action is produced by the friction at the contact line between the material and the sliding rotation of the two wheels (setup shown in Figure S8). During this process, the wheel creates a circular track. The abraded zone forms a circular band having a 10 mm width and a surface area of approximately 30 cm^2^.

For the liquid-mediated abrasion experiments, distilled water without detergents served as the liquid media. Conducting abrasion in liquid media was intended to represent abrasion events that could occur under wet conditions, such as during mopping of floors. Additional steps for abrading NC specimens in water are illustrated in Figure S8. The samples were fully immersed in water with ≈1 mm of overlying water. All abrasions were conducted at a speed of 6.28 rad/s (60 rpm) and 1000 g loading for 100 cycles. To evaluate the impact of the instrument vacuum being used, measurements were made using a 1% nanoAl_2_O_3_ PU coating system. The number of particles located at the abraded nanocoating surface was imaged with LSCM with or without the use of the vacuum, and no measurable difference in particle count was observed (Figure S9). The abrasion operation was performed in a nanoenclosure to avoid inhalation of released particles during the abrasion process.

**Figure 1:**
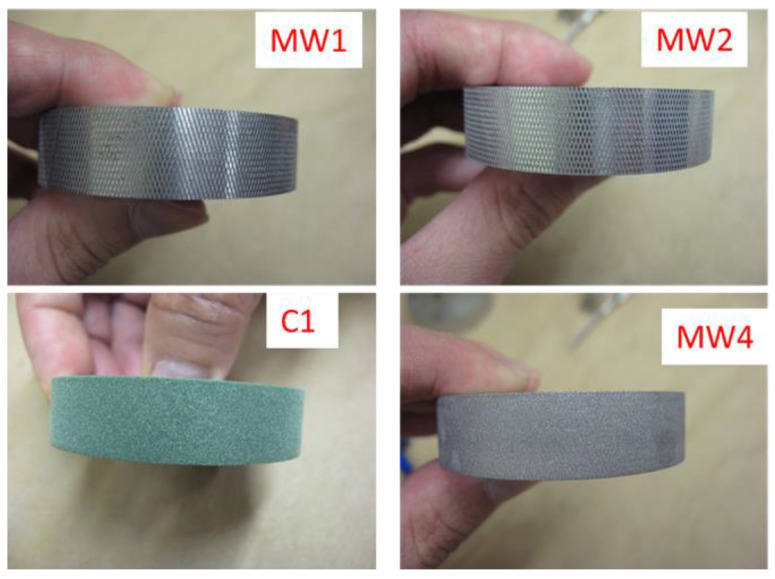
Side view of four of the five wheels used in this study. MW3 is not shown because a photograph was not taken before sandblasting to make MW4.

The following five wheels were evaluated in this study (shown in Figure 1):

- C1: Commercial abrasion wheels having a rubberized abrading surface (mild to medium abrading action); soft and smooth. Mechanical profiling data was not collected because the abrading surface was too soft.
- MW1: A stainless steel wheel (410 SS, which is easy to machine)-0.0015” (38.1 µm) deep cross patch (MW stands for metallic wheel).
- MW2: A stainless steel wheel (316 SS, chromium-nickel stainless steel with added molybdenum, highly corrosion resistant)-0.00075” (19 µm) deep cross patch.
- MW3: Obtained by polishing MW2 wheel with 120-grit sand paper.
- MW4: MW3 wheel sandblasted with silica-free coal slag blasting abrasives.

Pictures of four of the five wheels are displayed in Figure 1 (a picture of the MW3 wheel was not taken before it was sandblasted to make the MW4 wheel). All wheels have the same width (10 mm) and diameter (44.4 mm). The C1 wheel is a commercial wheel, while the other four stainless steel wheels that have specific surface roughness and patterns were fabricated at NIST. The surface patterns of the MW1 and MW2 wheels are similar, but with different surface roughness. Likewise, the surface patterns of the MW4 and the commercial C1 wheels are similar. Stainless steel was tested because regular steel, although harder, was found to corrode during and after abrasion in water. Surface profiles and root mean square (RMS) roughness values of these wheels are shown in Figure S10. The ranking of surface roughness of the five wheels is as follows: MW1 > MW2 > MW4 > C1 > MW3. After abrasion, these wheels form a circular band having a 12.5 mm width and a surface area of approximately 30 cm^2^ on the nanocoated specimens. All results on the number and size distribution of release particles accumulated on the nanocoated surfaces after abrasion were based on measurements in this circular abraded band.

### 2.4. Quantification of Released Particles on the Sample Surface

LSCM, operating in reflection mode, is a fast, microscopic technique suitable for imaging particles and larger clusters of particles on a specimen surface. When combined with image analysis, LSCM can provide quantitative information on the number and distribution of particle sizes ranging from 80 nm to 10 µm. Preliminary testing of this approach was performed on 300 nm monodisperse polystyrene spheres (Nanobead NIST Traceable particle size standard, 300 nm, Polysciences Inc., Warrington, PA, USA) and 50 nm silver nanoparticles synthesized as described in [42].

To confirm that the particles observed using LSCM on the sample surface after abrasion are mainly due to the particles generated by the abrasion process, adhesive tape was used to assess if there was a dramatic decrease in the quantity of particles present after applying and then removing the adhesive tape from the sample surface. A nanoAl_2_O_3_ polyurethane coating was tested using the following abrasion conditions: 60 rpm (6.28 rad/s), a loading of 1000 g, and 100 abrasion cycles. For this purpose, Scotch tape was applied on the abraded surface and rolled forward repeatedly 10 times with a tweezer at an applied normal load of approximately of 255 g ± 5 g (the normal load was measured with a hand-held balance). The adhesive tape was immediately removed, and both the abraded and adhesive tape surfaces were imaged and analyzed by LSCM at 150× magnification.

A measurement method is also under development to evaluate the airborne released particles using this abrasion method with the results showing that the number of released particles is close to the background concentration in the laboratory (data not shown).

## 3. Results and Discussion

### 3.1. Development of LSCM Method for Quantification of Release Particles on NC Surfaces

In general, the collected reflected light intensity is proportional to (particle size)^6^ × (difference in the index of refraction between the particle and polymeric matrix)^2^. The smallest spot size is 80 nm, which is the detection limit of the LSCM (the smallest spot size of 80 nm is equivalent to the length of one pixel) based upon the collecting optics in the microscope. However, the actual size of particles below 291 nm (diffraction limit of 543 nm light for 150× objective, numerical aperture of 0.95) cannot be fully resolved due to the diffraction limit of the reflected light. Nevertheless, particles with sizes between 80 nm and 291 nm can be identified and their sizes can be estimated. To demonstrate this, an example image of well-defined 300 nm polystyrene beads with a diameter smaller than the diffraction limit of the reflected light is shown in Figure S11. Nanoparticles with dimensions below the pixel size, 80 nm, can also still be identified but not estimated for size, since the bright spot originating from the particle will fill the entire pixel. An example image of 50 nm silver nanoparticles, below the 80 nm spot size, is shown in Figure S12. Particles smaller than 80 nm may still be identified using LSCM if the signal is sufficiently large and the particle is not in the vicinity of larger particles. It is possible that the particle number could be biased by not identifying particles or by identifying more than one particle occupying an 80 nm by 80 nm area as a single particle.

This study combined LSCM with image analysis to provide quantitative information on the number and size distributions of released particles on the sample surface having sizes > 80 nm. LSCM has several attributes that are attractive for the quantitative analysis of particles on the sample surface after abrasion, including fast speed; nondestructive, noncontact, and ambient operation; and analysis of a large sample area. In addition, LSCM has the ability to image xy slices at different depths (z-direction) into a material, depending on the material’s refractive index, which enable LSCM to be used for detection and quantification of the number of particles on nanocoatings with rough surfaces features (approximately greater than 6 µm). In contrast, other commonly used nanoparticle characterization techniques such as AFM and SEM usually collect images of particles in only one focal plane, making it more challenging to detect all of the released particles present on rougher nanocoating surfaces. The presence of released material on the sample surface was confirmed applying adhesive tape on the abraded surfaces, followed by LSCM imaging of both the tape and abraded surfaces; the tape was shown to remove 67% of the particles counted by LSCM.

A three-dimensional (3D) rendered image and 2D projection images in the X-Y and X-Z planes are also presented to illustrate the particle distribution inside the films before abrasion (Figure S7). To eliminate the strong reflection from the polymer-air surface, an oil lens with a magnification of 100× and a numerical aperture of 1.3 was also used to image the particle dispersion inside the nanocoatings.

**Figure 2:**
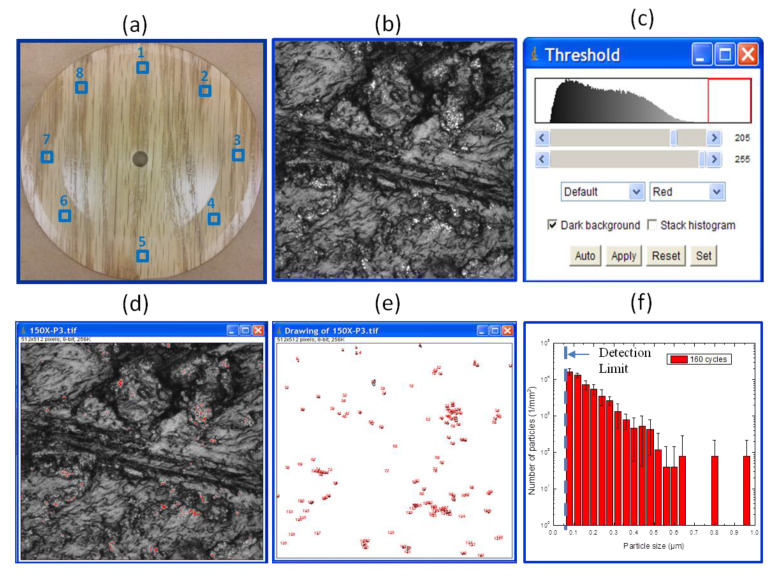
An example of using image analysis in laser scanning confocal microscopy (LSCM) to obtain the number and size distribution of surface release particles generated by abrasion of a nanocoating (NC). (**a**) a picture of an abraded specimen showing the 8 different positions where the LSCM images were taken for analysis, (**b**) a 150× LSCM image of one representative abraded surface, (**c**) the grey level threshold setting, (**d**) the highlighted particles within the image as defined by the grey level threshold in (**c**), (**e**) the spatial distribution of highlighted particles after background removal, and (**f**) a bar plot of the total particles counted for each particle size bin with bins ranging from 0.08 µm up to 1 µm. Error bars in (**f**) represent one standard deviation from the average of eight measurements from four samples (each sample was analyzed in two of the eight locations shown in (**a**)). The dashed line in (**f**) represents the detection limit for this LSCM method as described in the text.

LSCM images of the abraded samples were taken at two magnifications 5× and 150×, with an optical slice (*z*-step) of 2 µm for 5× and 0.1 µm for 150×, respectively, at the locations shown in Figure 2a. LSCM graphs reported in this study are 2D projection images using Zeiss confocal software. Image analysis was performed using the freeware *ImageJ* (Wayne Rasband, NIH, Bethesda, MD, USA, https://imagej.net/ImageJ). An example of this unique methodology is illustrated in Figure 2.

### 3.2. Surface Release Particles by Dry Abrasion

**Figure 3:**
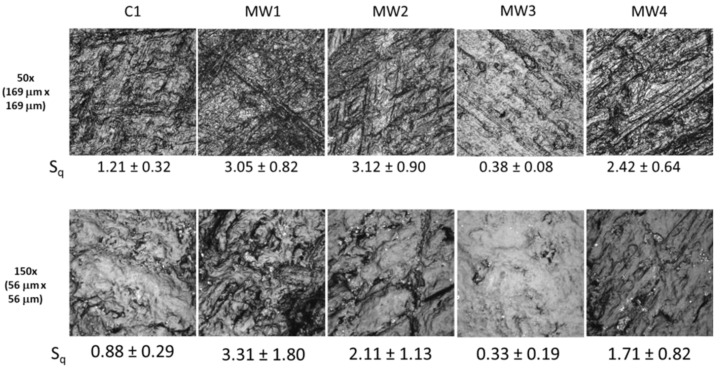
LSCM images taken at 50× and 150× magnifications on dry abraded NC surfaces by five different wheels. Abrasion was performed using the following parameters: 6.28 rad/s (60 rpm) speed, 1000 g applied force, 100 cycles, and the vacuum turned off. S_q_ (Root mean square (RMS) surface roughness values), in μm, are also included below each image for comparison. The results are mean ± one standard deviation value from eight measurements (two measurements were made on each of four specimens).

To assess the most suitable wheel type for use in abrasion studies, four metallic wheels (MW1, MW2, MW3, and MW4) with different surface patterns and roughness values, and one commercial wheel composed of rubber abrasives that is widely used for evaluation of coatings, C1, were evaluated. Representative LSCM images were taken at two different magnifications for NC surfaces abraded using the five wheels (Figure 3). The bright regions in an LSCM image are due to greater light reflection from materials that are at or above the surface; and the darker regions represent less-reflective materials, or those that are below the surface. In general, metallic particles reflect more light than polymers in a given plane as described in the SM. Thus, the bright particles observed in these images are likely loose particles generated by the abrasion process, i.e., surface release particles (release particles deposited on abraded surfaces).

The surface roughness values of NCs abraded by the five wheels (using the LSCM 169 µm × 169 µm [50×] and 56 µm × 56 µm [150×] scan sizes) are presented in Figure 3. It should be noted that larger scan size images generally represent the average surface roughness better than smaller scan size images, because roughness values obtained within smaller dimensions do not capture the larger surface features [43]. This is why roughness values differ depending upon magnification used. Despite a marked difference in their surface profiles, the MW1 and MW2 wheels produced essentially the same abraded surface roughness. The roughness values obtained after abrasion with the MW4 wheel were lower than those of MW1 or MW2. All MW abrasion wheels produced rougher surfaces than the C1 abrasion wheel. Furthermore, the MW3 wheel produced the smoothest abraded surface, as predicted, based on the surface profile shown in Figure S10. Surface roughness values of NCs abraded by the five wheels had the following decreasing order: MW2 ≈ MW1 > MW4 > C1 > MW3.

While the detection limit of LSCM hinders quantification of nanoparticles smaller than 80 nm, previous studies have shown little evidence of released particles from abrasion with diameters smaller than 100 nm [24,25,27,44,45]. The size distribution of airborne particles emitted by an abraser for polymer nanocomposites containing nanoZnO [25], nanoTiO_2_ [27], and MWCNT [24] has been reported to include particle sizes ranging from 0.1 µm to 50 µm. Furthermore, the majority of particles released during abrasion of polymer coatings and paints were found to contain nanoparticles embedded in the polymer.

**Figure 4:**
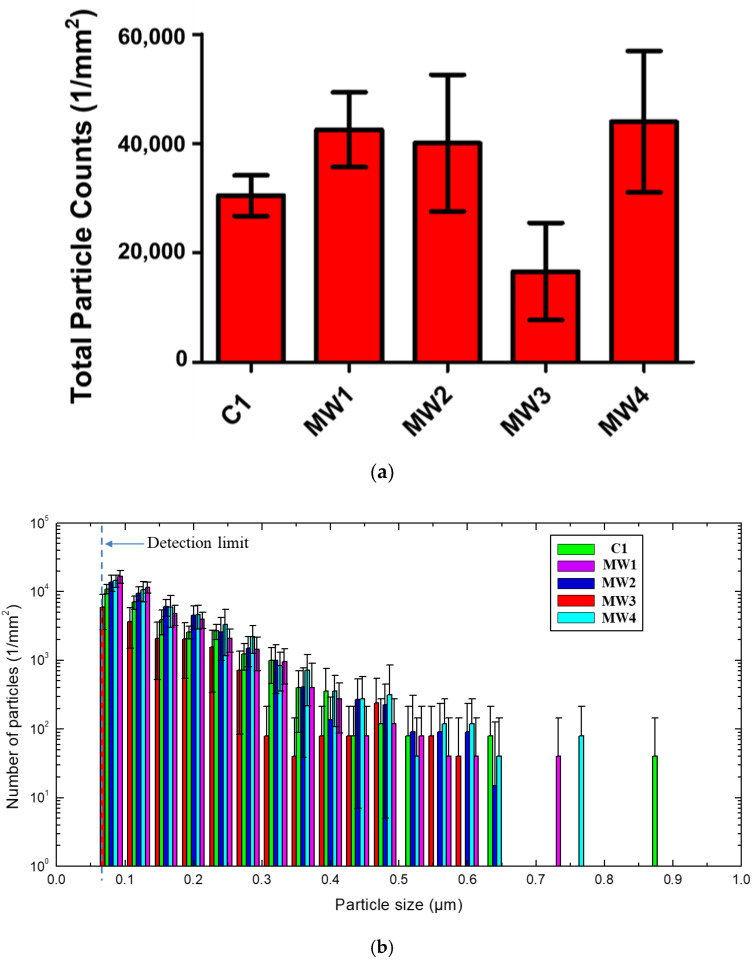
Total number (**a**) and size distribution (**b**) of released particles on the sample surface per 1 mm^2^ abraded area of NC for the five wheels. The data were obtained using 150× images. The results are the average of eight measurements (two measurements were made in each of four replicate specimens), and the error bars represent one standard deviation. The dashed line in part (**b**) represents the detection limit for this LSCM method. Note that the number of particles on the vertical axis in part (**b**) is expressed with a logarithmic scale.

The effect of wheel type on the number and size distribution of surface release particles for NCs was investigated (Figure 4) using the images at 150×. The majority of the released particles on the NC surface had a size ranging from 0.08 µm to 0.6 µm, with 95 ± 1 percent between 0.08 µm and 0.3 µm (Figure 4b). Figure 4b shows that the number of surface release particles generally decreased with increasing particle size, with the largest number for 0.08 µm and the smallest for 0.6 µm to 0.9 µm.

MW1, MW2, and MW4 generated close to the same total number of surface release particles for the NCs, while the two smoother wheels (MW3 and C1) produced fewer particles (Figure 4), indicating that the number of surface release particles depends strongly on the type of abrasive wheel. The smoothest wheel (MW3) generated the fewest surface release particles (only one third of the amount released from MW4). In addition, the coefficients of variation (standard deviation divided by the mean) of wheels C1, MW1, MW2, MW3, and MW4 were 0.122, 0.161, 0.312, 0.536, and 0.294, respectively. No data on the unabraded NC surface is presented here because there are only a few particles on the unabraded NC surface (Figure S7a). Wheel MW3 had the highest coefficient of variation, indicating that it was the least reproducible wheel to use for nanoparticle release studies. Wheels MW2 and MW4 also produced relatively high coefficients of variation, but were less variable than MW3. Undoubtedly, the abraded surface features, the relative grey level threshold selection in the image analysis, and the variability of particle distribution on the surface during the repeated abrasion also contributed to the high standard deviation values. It is also noted that surface release particles tend to accumulate on the edge of the circular abraded ring and redistribute on the abraded surfaces after each cycle. Therefore, further investigation of the profile/surface roughness of the wheel(s) and abrasion parameters (number of cycles, loads) is needed to generate reproducible release of particles from NC by abrasion.

### 3.3. Effects of Wheel Type on Surface Release Particles by Abrasion in Liquid

**Figure 5:**
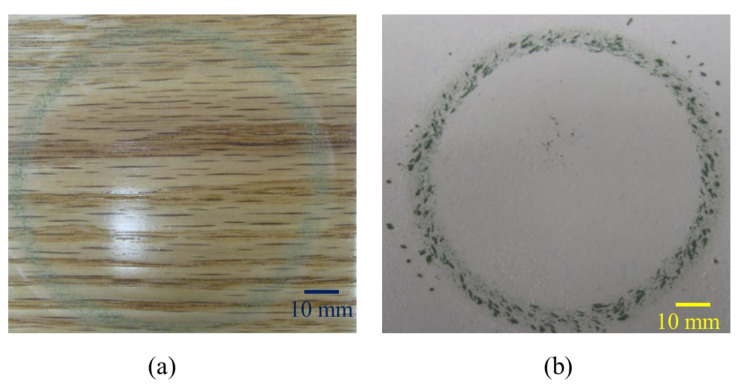
Photographs of (**a**) a 1% nanoAl_2_O_3_ coating on polyurethane and (**b**) a TiO_2_ nanopaint on drywall after abrasion with a commercial C1 wheel. Green particles having the same color as the abrasion wheel were observed on the circular tracks of the both samples, suggesting that the material was released by the wheel itself. The instrument vacuum was turned off during these measurements.

To achieve better statistics for release particles and mass loss analyses, a wheel that generates a higher number of released particles is preferred. In this case, MW2 and MW4 were chosen for additional analysis with abrasion of the NC specimens in water. MW1 was not selected because it was made of less corrosion-resistant stainless steel, and MW1 and MW4 have similar roughness and surface patterns. The MW3 wheel was not used for additional testing because it had the highest coefficient of variation value. The C1 wheel was not used because it was shown to release particles from itself during abrasion (Figure 5).

**Figure 6:**
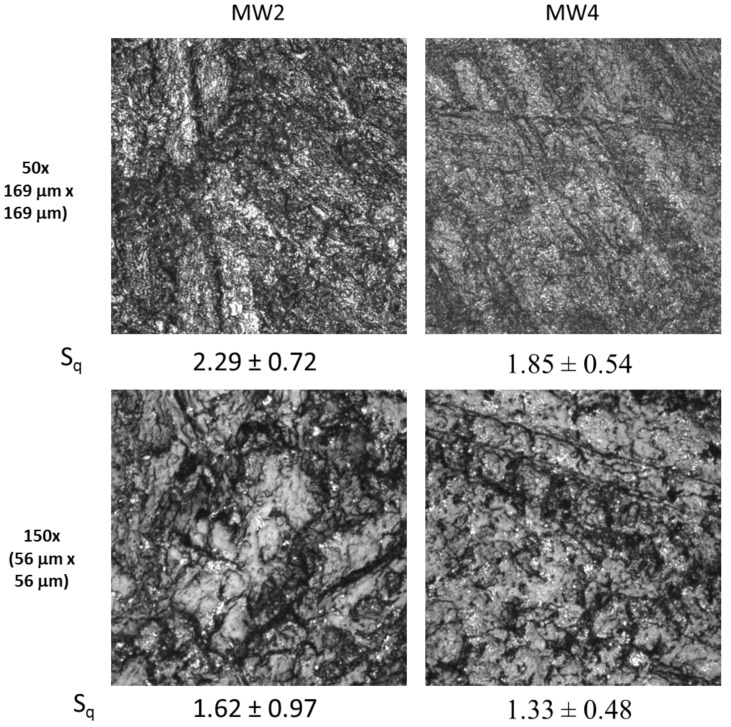
LSCM images taken at two magnifications for wet abrasion of NC using two different metallic wheels (MW2 and MW4). Abrasion parameters: 6.28 rad/s (60 rpm) speed; 1000 g applied force, and 100 cycles. S_q_ (RMS surface roughness values), in μm, are also included below each image for comparison. The results are mean ± one standard deviation value from eight measurements (two measurements were made on each of four specimens).

**Figure 7:**
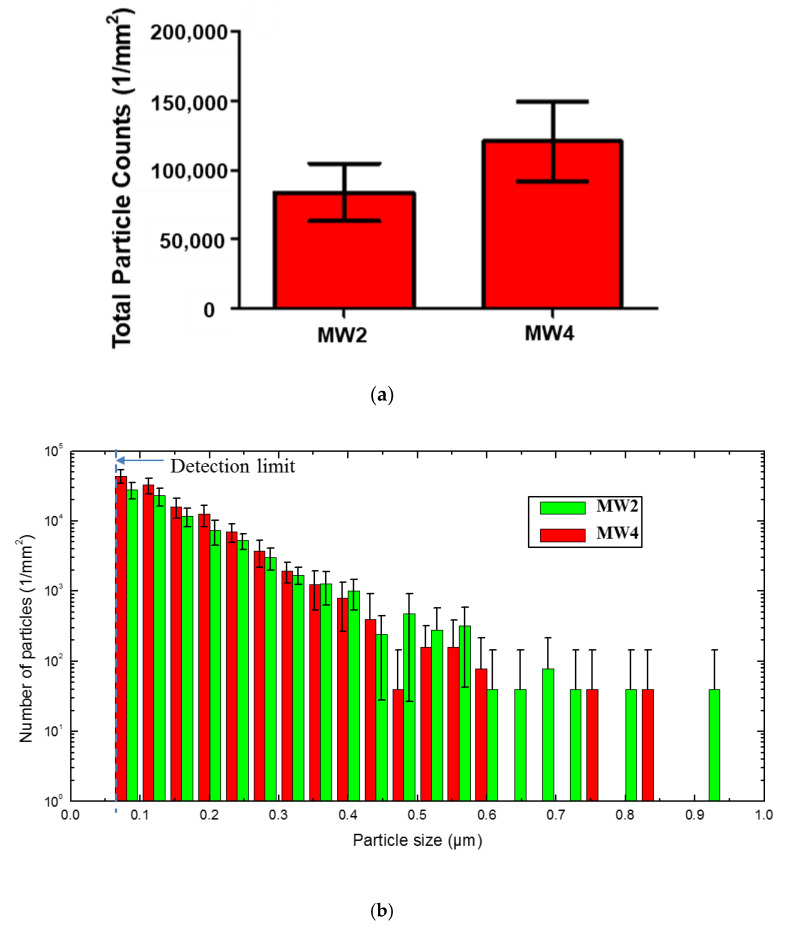
Total number (**a**) and size distribution (**b**) of released particles on the sample surface per 1 mm^2^ abraded area of NC for MW2 and MW4. The data were obtained using 150× images. No data on the unabraded NC surface is presented here because there are only a few particles on the unabraded NC surface (Figure S7a). The results are the average of eight measurements (two measurements were made in each of four specimens), and the error bars represent one standard deviation. The dashed line in part (**b**) represents the detection limit for this LSCM method. Note that the number of particles on the vertical axis in part (**b**) is expressed with a logarithmic scale.

Figure 6 displays representative LSCM images obtained at two different magnifications for NC surfaces abraded in water using two different metallic wheels, MW2 and MW4 (for surface profile and roughness of these two wheels, see Figure S10). The MW4 wheel released more particles of all sizes than the MW2 wheel and 40 percent to 50 percent more particles with sizes <2.0 μm (Figure 7). For both wheels, the results show that the majority of particle sizes generated by wet abrasion were between 0.08 µm and 0.3 µm, a result similar to what we observed for dry abrasion. However, under the same parameters, and for all particle sizes, wet abrasion yielded two times to three times more surface release particles, compared to dry abrasion. As shown in Figure S2 and Table S1, the ultimate tensile strength (at break) and modulus values of the NC, after water saturation, were less than half and one quarter, respectively, of their initial values. This substantial decrease in mechanical properties helps explain the greater number of released particles during abrasion of NC in water, where the samples were fully immersed. Additionally, the coefficients of variation for surface release particles by wet abrasion are lower than those produced by dry abrasion. 

## 4. Conclusions

A substantial number of released particles were detected on the NC surface after abrasion in both water and in air, with 2 to 3 times more particles generated during wet abrasion (Figure 3, Figure 4 and Figure 6). The sizes of surface release particles of both nanocoatings abraded in air and in water varied between 0.08 µm and 0.6 µm, with 95 ± 1 percent of the particles observed in the 0.08 µm to 0.3 µm range. For both conditions, the number of surface release particles decreased with increasing size. Additional work is needed to investigate how to capture these released particles, including both the surface-located and airborne particles, for further characterization or use in other experiments (e.g., toxicity assays).

Overall, MW2 and MW4 were both suitable for abrasion under wet and dry conditions. The other wheels had substantive limitations: the MW1 wheel was corrodible under wet conditions; the MW3 wheel yielded the most variable results; and the C1 wheel released particles itself during abrasion, as shown in Figure 5. Although the MW4 yielded a higher total number of released particles on the sample surface during abrasion under wet conditions, the MW2 wheel was selected as the optimal wheel because it was straightforward to produce, which is an important factor in choosing an abrasion wheel for a standardized method. In contrast, MW4 required an additional sand-blasting step, which offsets its advantage of releasing more particles.

The results described in this study can help advance the science in the nanoparticle release area by highlighting potential pitfalls of using some wheels (e.g., the potential for release of particles from the wheels themselves which could bias the results), guiding authors toward using robust wheels for future studies, and providing a method for quantifying surface-located particles using LSCM. The methods described in this Communication can help support other researchers in future studies to investigate the mechanisms involved for the release of different nanofillers from NC specimens under different uses. In addition, researchers may now use the information provided in this manuscript to generate particles with suitable abrasion wheels for use in ecological and health risk assessment.

## Supplementary Materials

The following are available online at https://www.mdpi.com/2079-4991/10/8/1445/s1, Figure S1. (a) Three 9.6 cm diameter discs having a 6.25 mm hole in the center milled from a wood panel; (b) 9.6 cm diameter discs inserted in a three circular perforation Teflon mold, and (c) liquid coating applied to the wood discs using a draw down applicator to generate nanocoated wood disc specimens used for abrasion in air and water, Figure S2. Representative stress-strain curves of NC before (upper) and after immersion in water for 1 d (lower), Figure S3. Representative TGA curve of NC using air as the carrier gas and a heating rate of 10 °C/min, Figure S4. EDS spectrum of the surface of non-abraded NC samples. The scale bar is 10 µm, Figure S5. SEM image (a) and four EDXS spectra (b) from a NC sample cross section (the locations where the spectra were collected are indicated in the SEM image), Figure S6. AFM images of unabraded NC replicates at three different magnifications. For each AFM pair, the height image is on the left and the phase image is on the right. The image sizes are included below each image, Figure S7. 2D LSCM images of unabraded NCs obtained using (a) air lens in X-Y project @ 150× (b) 3D images using an oil immersion lens (100×) (c) in X-Y projection and (d) in X-Z projection. The image sizes are included below each image, Figure S8. Instrument setup for abrasion in water:(a) accessory for abrasion in water; (b) a coated wood specimen (dia. = 9.6 cm) in the accessory; (c) a specimen on the accessory mounted on the rotary abraser, and (d) same as (c) but with addition of distilled water so that there was ≈1 mm of overlying water, Figure S9. Effect of vacuum on the number and size distribution of surface-located released particles of the 1% nanoAl_2_O_3_ polyurethane floor coating; (a) with the vacuum on and (b) with no vacuum. A C1 wheel was used, and the abrasion parameters were the following: speed: 60 rpm, loading of 1000 g, number of cycles:100. Error bars represent one standard deviation. All measurements were conducted away from green particle cluster areas, Figure S10. The surface profiles and roughness values of three different metallic wheels were measured with a profilometer: MW1, MW2 (including MW3), and MW4. Here Rq is the RMS (root-mean-square) roughness, Figure S11. LSCM image at 50× (169 µm × 169 µm) of 300 nm diameter polystyrene beads. Despite being below the diffraction limit (308 nm) of the incident/reflected 543 nm light, these beads are detectable and their size can be estimated to be 300 nm. The size becomes more challenging to estimate with decreasing size but still can be estimated down to the smallest spot size of the detector, which is 80 nm, Figure S12. LSCM image (150×, 56 µm × 56 µm) (top) compared to SEM image (56 µm × 56 µm) of 50 nm particles (bottom), below the 80-nm spot size (or pixel size) possible with the LSCM detector. Particles with sizes below 80 nm can be observed with LSCM, but show up as bright spots that fill a pixel. With a lot of particles in close proximity to each other, neighboring pixels can give the illusion of larger particles being present, Table S1. Relevant initial properties of NC used in this study. The uncertainties represent one standard deviation from at least 3 specimens.

## Disclaimer

Mention of trade names or commercial products does not constitute endorsement or recommendation for use, nor does it imply that alternative products are unavailable or unable to be substituted after appropriate evaluation. Certain commercial entities, equipment, or materials may be identified in this document in order to describe an experimental procedure or concept adequately. Such identification is not intended to imply recommendation or endorsement by the National Institute of Standards and Technology or the U.S. Consumer Product Safety Commission nor is it intended to imply that the entities, materials, or equipment are necessarily the best available for the purpose. The findings and conclusions in this paper are those of the authors and do not necessarily represent the views of the U.S. NIST or the U.S. Consumer Product Safety Commission.
