# Supplementary material for: Selection of an Optimal Abrasion Wheel Type for Nano-Coating Wear Studies under Wet or Dry Abrasion Conditions
Source: Nanomaterials (Basel). 2020 Jul 24;10(8):1445. doi: 10.3390/nano10081445 (PMC7466352; doi:10.3390/nano10081445)
Supplement: Supplementary file 1 [file nanomaterials-10-01445-s001.pdf]

nanomaterials

Communication

Selection of an Optimal Abrasion Wheel Type for Nano-Coating Wear Studies under Wet or Dry Abrasion Conditions

Li-Piin Sung [1,*], Yu-Fan Chung [1], David G. Goodwin, Jr. [1], Elijah J. Petersen [2,*], Hsiang-Chun Hsueh [1], Paul Stutzman [1], Tinh Nguyen [1] and Treye Thomas [3]

1 Engineering Laboratory, National Institute or Standard and Technology (NIST), Gaithersburg, MD 20899, USA; moonlist31@gmail.com (Y.-F.C.); david.goodwin@nist.gov (D.G.G.J.); d9631837@oz.nthu.edu.tw (H.-C.H.); paul.stutzman@nist.gov (P.S.); tnguyen6718@outlook.com (T.N.)
2 Materials Measurement Laboratory, NIST, Gaithersburg, MD 20899, USA
3 Office of Hazard Identification and Reduction, U.S. Consumer Product Safety Commission, Bethesda, MD 20814, USA; tthomas@cpsc.gov
* Correspondence: li-piin.sung@nist.gov (L.-P.S.); elijah.petersen@nist.gov (E.J.P.); Tel.: +1-3019756737 (L.-P.S.); +1-3019758142 (E.J.P.)

1. Supplemental Methods

1.1 Preparation of nanocoating (NC) specimens for abrasion

The wood substrates used in this study were commercial hardwood plywood panels consisting of a poplar core and red oak veneer faces. Each wood panel had 17.8 cm × 35.5 cm × 5.35 mm (7 inch × 14 inch × 1/5 inch) dimensions and was cut into three 9.6 cm diameter circular disks (the necessary size for the liquid abrasion accessary) that were still attached to the panels, as shown in Figure S1a. The center of each disc was drilled to provide a hole having a diameter of 6.25 mm, which was slightly larger than the diameter of the specimen support of the liquid abrasion accessory. After separating the discs from the wood panel by a cutter, the discs were then inserted in a three circular perforation Teflon mold (Figure S1b). The diameter of the perforation was slightly larger than that of the disc so that the disk could fit snugly in the perforation. The depth of the perforation was about 5.35 mm, which was approximately equal to the thickness of the wood panel. Care was taken so that the surface of the wood discs was at approximately the same level with that of the Teflon mold. In this way, the thickness of the nanocoating on the wood substrate was approximately the same for all specimens. Three holes were drilled on the bottom of each mold's perforation to facilitate dislodging of the coated discs after applying the NC.

Nanocoatings were applied on the wood discs in the Teflon mold using the drawdown technique (Figure S1c). For this process, a volume of viscous liquid coating or paint was spread on one end of the substrate (wood discs in the Teflon mold in this case), and the liquid was drawn down slowly and steadily using a drawdown applicator. This common technique provides a uniform film thickness of polymeric materials on a substrate. After the first coating, discs were removed from the Teflon mold and dried for 8 h at ambient conditions (24 °C and ≈ 50% relative humidity). The coated discs were then inserted back to the mold, and another layer of NC was applied to provide a second layer. A two-layer coating was used in this study because the wood substrate absorbs a substantial amount of water-based coating. If one-layer specimens were used, only a very thin layer of NC would be left on the wood surfaces. This would result in an unavoidable removal of the wood substrate during the abrasion of the NC. The nanocoated wood disks were then cured for two weeks at ambient conditions, as recommended by the manufacturers.

1.2 Specimen preparation for 1% nanoAl_2O_3 polyurethane floor coating and 1.2% nanoTiO_2 latex interior paint

In addition to the NC described in 1.1, a commercial water-based polyurethane (PU) floor coating with 1% nanoAl_2O_3 and a 1.2% nanoTiO_2 latex interior paint were also prepared for additional

analyses: vacuum study, evaluation of removal of LSCM-detected particles after Scotch tape was applied to the sample surface, and evaluation of particle release from the C1 wheel onto the sample surface. Note, all mass percentages are based on the mass of the polymer matrix; all percentages for components of the nanocoatings refer to mass fractions. The nanofilled PU had Al_2O_3 nanoparticles that were 20 nm in diameter. The latex paint was a typical interior wall paint containing 30% nepheline syenite tint base (aluminum silicate filler). The size of the nepheline syenite tint base particles was between 5 μm and 10 μm. The latex paint contained nanoparticles (nanoTiO_2) that had a diameter of 30 nm. Information was provided by the commercial supplier (BYK USA Inc., Wallingford, CT, USA) for the generic names, nanoparticle mass loadings, and nanoparticle size, but other properties about the chemical composition and physical properties of the PU floor coating and interior latex paint were not available.

The nano-filled PU floor coatings for vacuum studies (1 % by mass Al_2O_3) were applied by brush on an oak wood substrate (6.25 mm thickness) and consisted of four separate layers (four coats). Each layer was allowed to air dry for 8 h before the next layer was applied, a procedure typical for water-based coating products sold in the consumer market for flooring finishes. The substrate was either one-piece or multiple 25 mm wide strips. The nano-filled latex paints were applied on a drywall substrate by roller and consisted of four layers with 4 h of air drying time between the layer applications. The drywall was a composite assembly having a thickness of 11 mm (7/16 inch), which consists of a 10-mm thick gypsum board sandwiched between two 0.5 mm thick paper boards.

2. Supplemental Results

2.1. Properties of the NC Surface before Abrasion

Representative stress-strain curves of the NC are presented in Figure S2. This material shows typical properties of a plastic material, i.e., it goes through a yield region and a long elongation before breaking. The modulus of elasticity of each material was obtained from the slope of the early portion of their respective curves, and was (325 ± 15) MPa.

The effect of water on the stress-strain behavior of the NC is also shown in Figure S2. The lower curve is from a NC that was immersed in distilled water for 24 h. This time period was required to saturate the 250 μm thick NC free film (see section 1.1); preliminary experiments measuring the rate of water uptake by the NC indicated that a steady-state (i.e., saturation) had been reached prior to 24 h.

2.2. Inorganic Content and Chemical Composition of the NC

The amount of inorganic materials, including nanoparticles, in the NC samples were determined by TGA. Because the inorganic content in the NC was small, an expanded TGA curve (inset) was included for this material (Figure S3). The mass losses between 100 °C and 300 °C were due to residual water/solvent and low molecular mass materials, such as plasticizers, surfactants, etc. The high molecular mass polymer chains in both materials started to decompose (marked by a decrease in the sample mass) around 300 °C due to combustion in air and should be completed removed by 800 °C. The mass loss was rapid between 300 °C and 500 °C for the NC. Further, the weight loss of the NC did not reach a constant value until around 650 °C, and loss of some inorganic material may have occurred before this temperature was reached. At 800 °C, which is not high enough to cause Al in the metal-oxide Al_2O_3 to be vaporized (in the TGA curves displayed in Figure S3), the total Al content (which may contain other inorganic content such as SiO_2) in NCs was (0.26 ± 0.01) percent. Because only nanoparticles were assumed to be present in the NC, the total inorganic content represents the total amount of nanoparticles in this material.

To identify chemical composition of the inorganic materials in the NC, Energy Dispersive X-ray Analysis (EDS) with scanning electron microscopy (SEM) was employed to characterize an unabraded sample. EDS analysis of the sample surface without treatment (Figure S4) and from the cross-section (Figure S5) showed detectable concentrations of Si and Al, a result in alignment with

the manufacturer's specification that Al particles had been used. The source of Si is unclear due inclusion of proprietary ingredients in the commercial product

2.3. Surface and Sub-surface Morphology of the Nanocoating Specimens

Surface morphology and roughness play an important role in the abrasion behaviors of polymeric materials. The surface morphology of NC specimens before abrasion was characterized with AFM and LSCM, and the results are displayed in Figures S6 and S7, respectively. Figure S6 shows both topographic and phase AFM images of NC specimens at three different magnifications. AFM phase imaging is a useful technique for distinguishing domains that have different mechanical and chemical properties, such as inorganic nanoparticles in polymer matrices. The phase images show the presence of particles on the surface of the NC specimens, with some particles having nanoscale dimensions.

Figure S7 displays surface morphology and subsurface features in 2D and 3D LSCM images. LSCM images of the NC surface before abrasion obtained with oil immersion lens (Figure S7) shows some large particles on the NC surface, and many large agglomerates near the surface.

Table S1. Relevant initial properties of NC used in this study. The uncertainties represent one standard deviation from at least 3 specimens.

Property	Measured Values
Specimen thickness, µm	253 ± 16
Density, g/cm^3	1.15
Young modulus, MPa	325 ± 15
Young modulus<br>water saturated, MPa	71.4 ± 4.0
Tensile strength, MPa	14.35 ± 0.37
Tensile strength<br>water saturated, MPa	5.59 ± 0.43
Glass transition temperature (T_g), °C	87.5 ± 1.0
Inorganic Solid content (TGA), %	0.26 ± 0.01

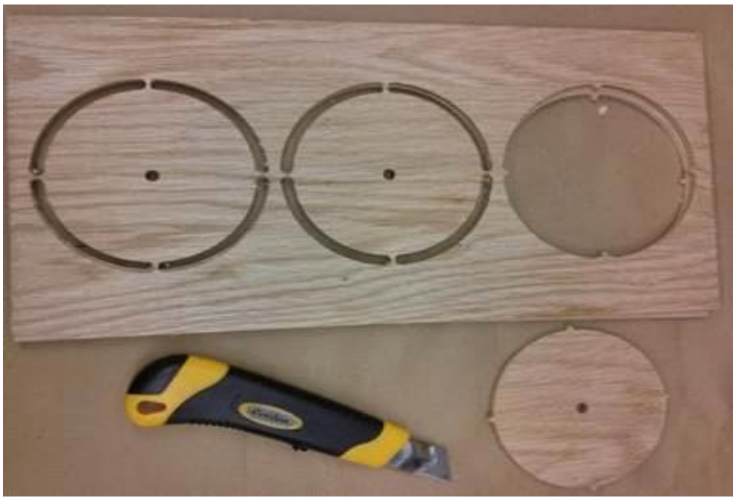

(**a**)

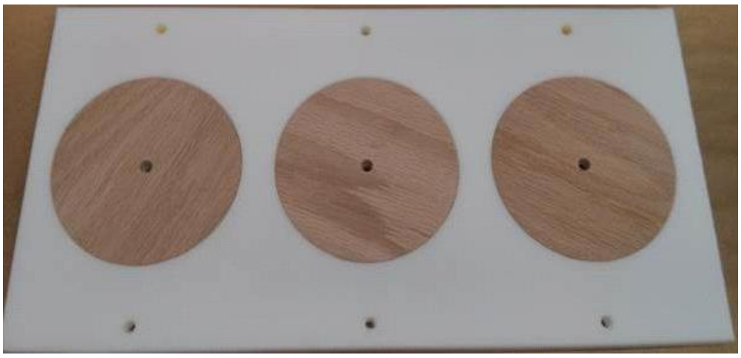

(**b**)

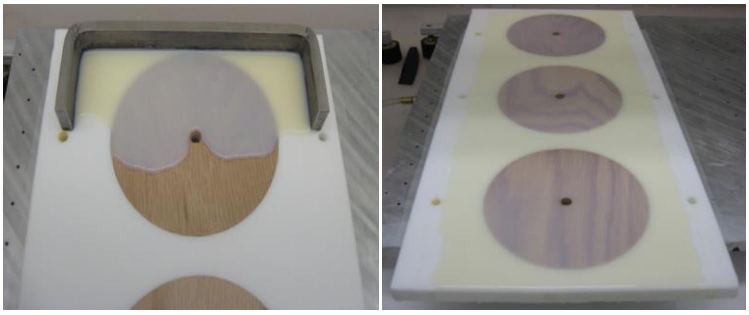

(**c**)

Figure S1. (**a**) Three 9.6 cm diameter discs having a 6.25 mm hole in the center milled from a wood panel; (**b**) 9.6 cm diameter discs inserted in a three circular perforation Teflon mold, and (**c**) liquid coating applied to the wood discs using a draw down applicator to generate nanocoated wood disc specimens used for abrasion in air and water.

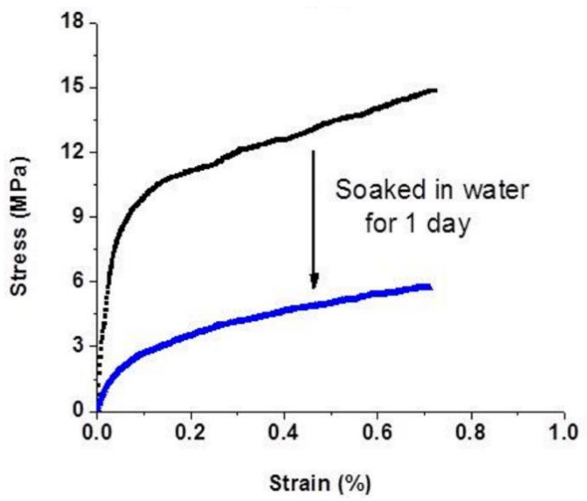

Figure S2. Representative stress-strain curves of NC before (upper) and after immersion in water for 1 d (lower).

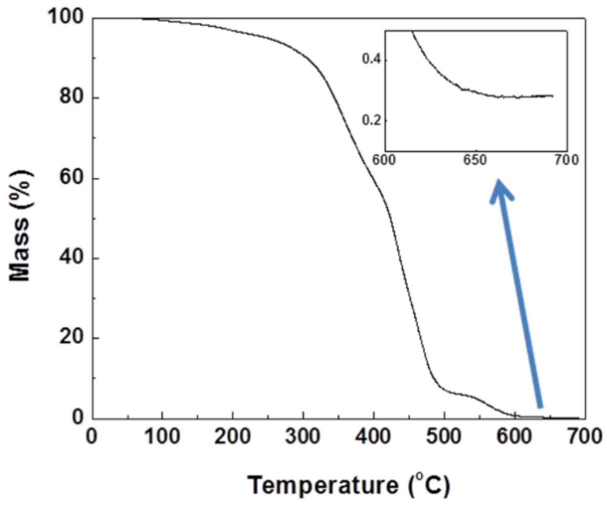

Figure S3. Representative TGA curve of NC using air as the carrier gas and a heating rate of 10 °C/min.

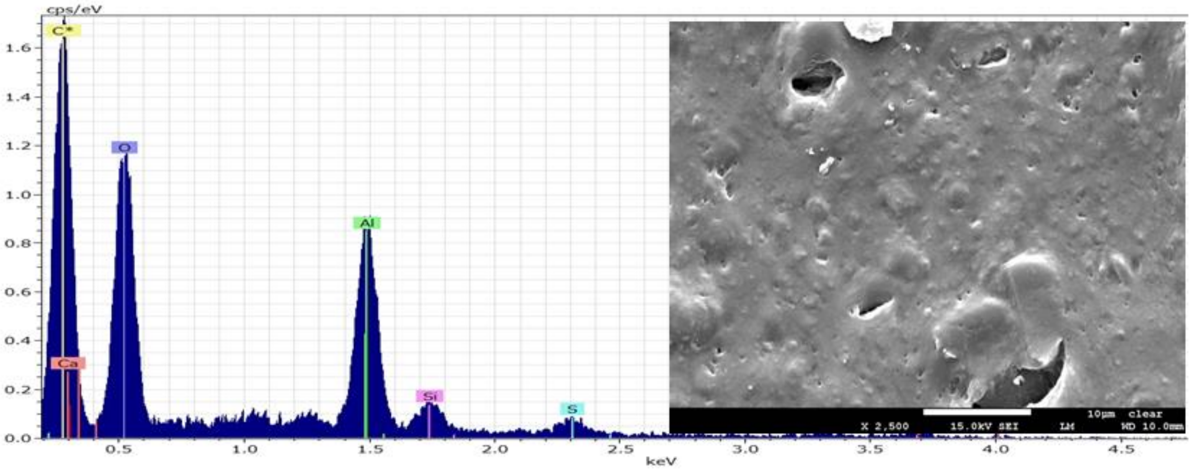

Figure S4. EDS spectrum of the surface of non-abraded NC samples. The scale bar is 10 μm.

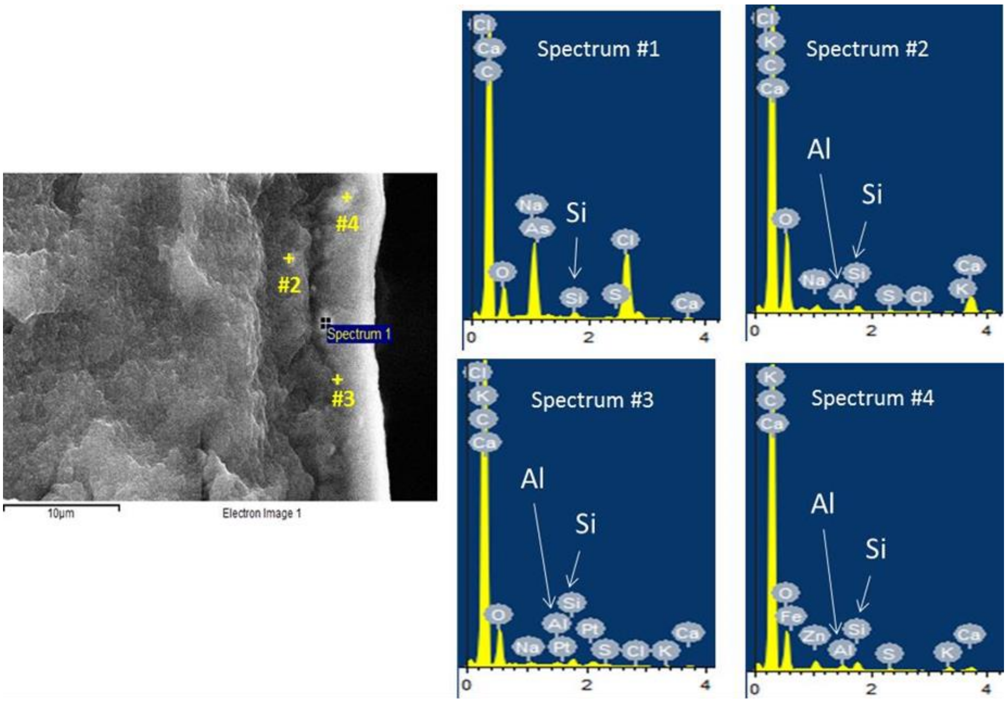

(a) (b)

Figure S5. SEM image (**a**) and four EDXS spectra (**b**) from a NC sample cross section (the locations where the spectra were collected are indicated in the SEM image).

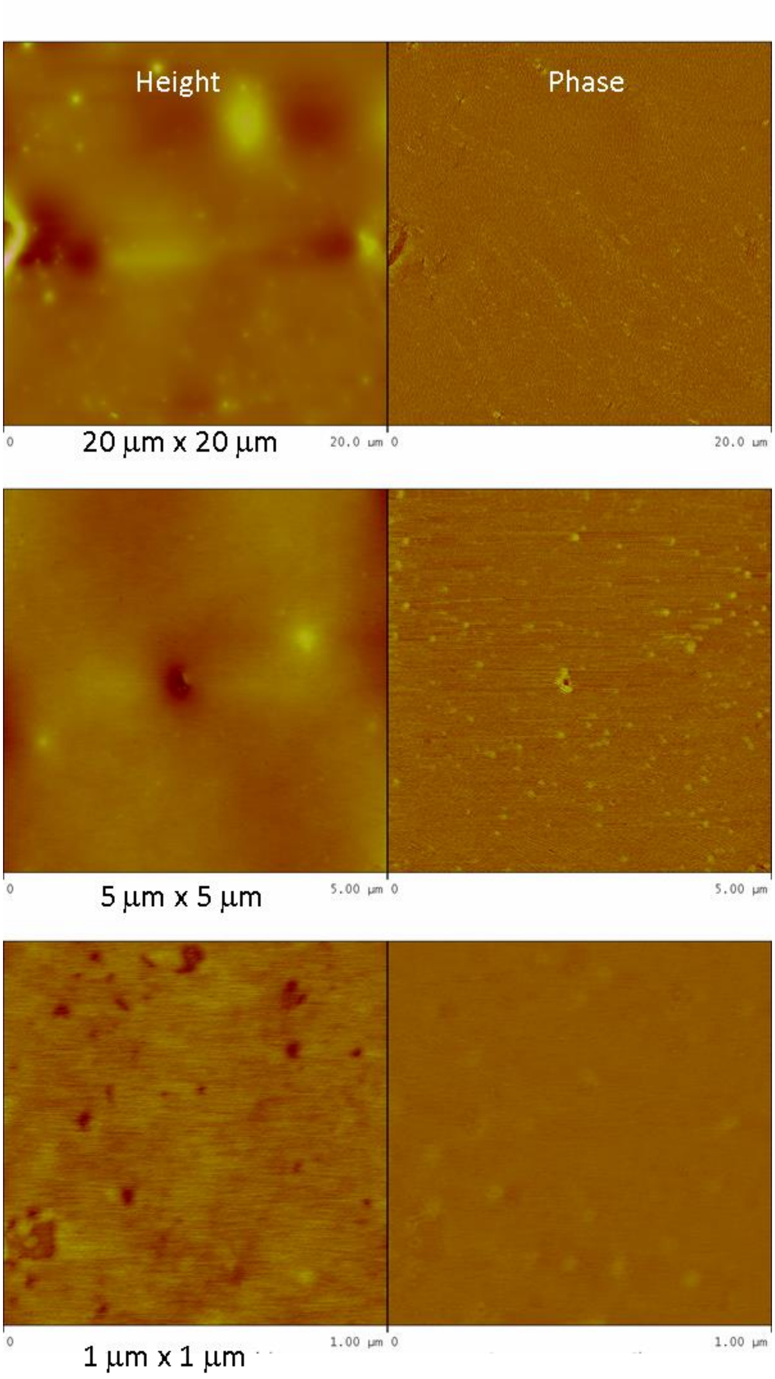

Figure S6. AFM images of unabraded NC replicates at three different magnifications. For each AFM pair, the height image is on the left and the phase image is on the right. The image sizes are included below each image.

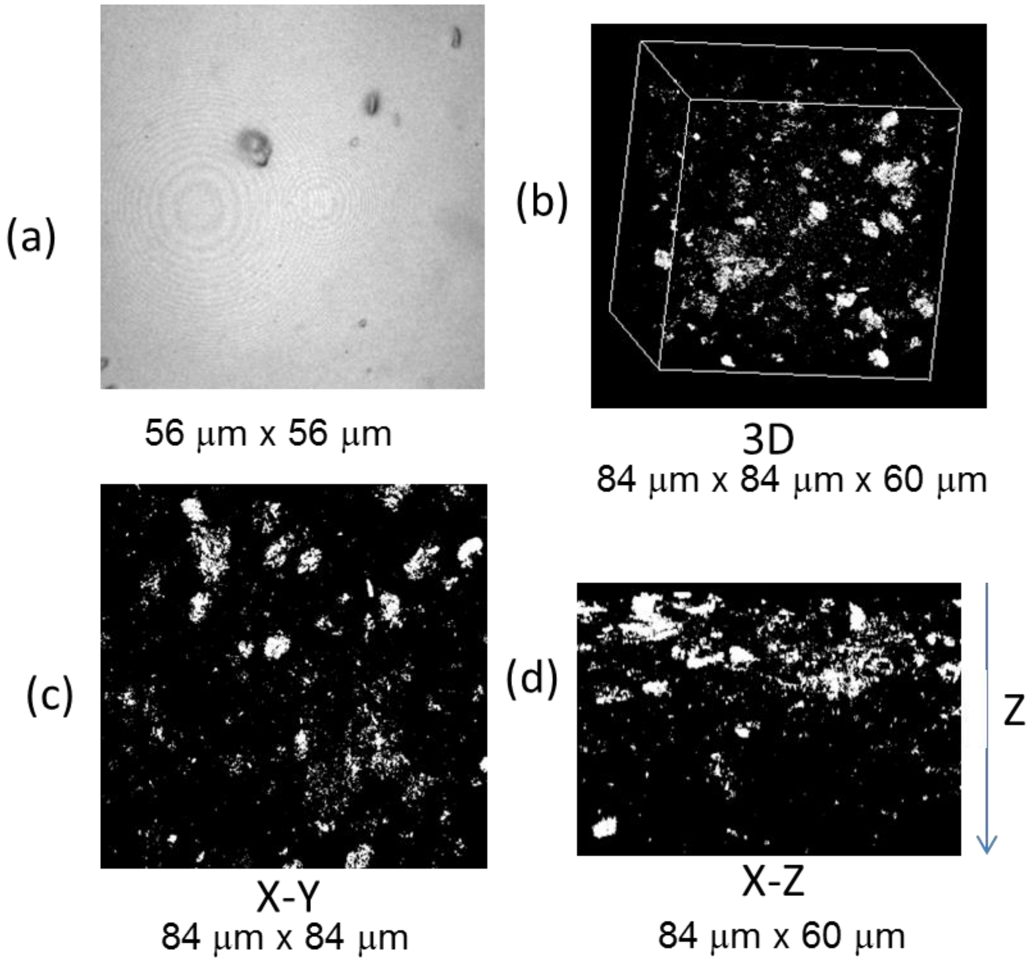

Figure S7. 2D LSCM images of unabraded NCs obtained using (**a**) air lens in X-Y project @ 150×, (**b**) 3D images using an oil immersion lens (100×), (**c**) in X-Y projection, and (**d**) in X-Z projection. The image sizes are included below each image.

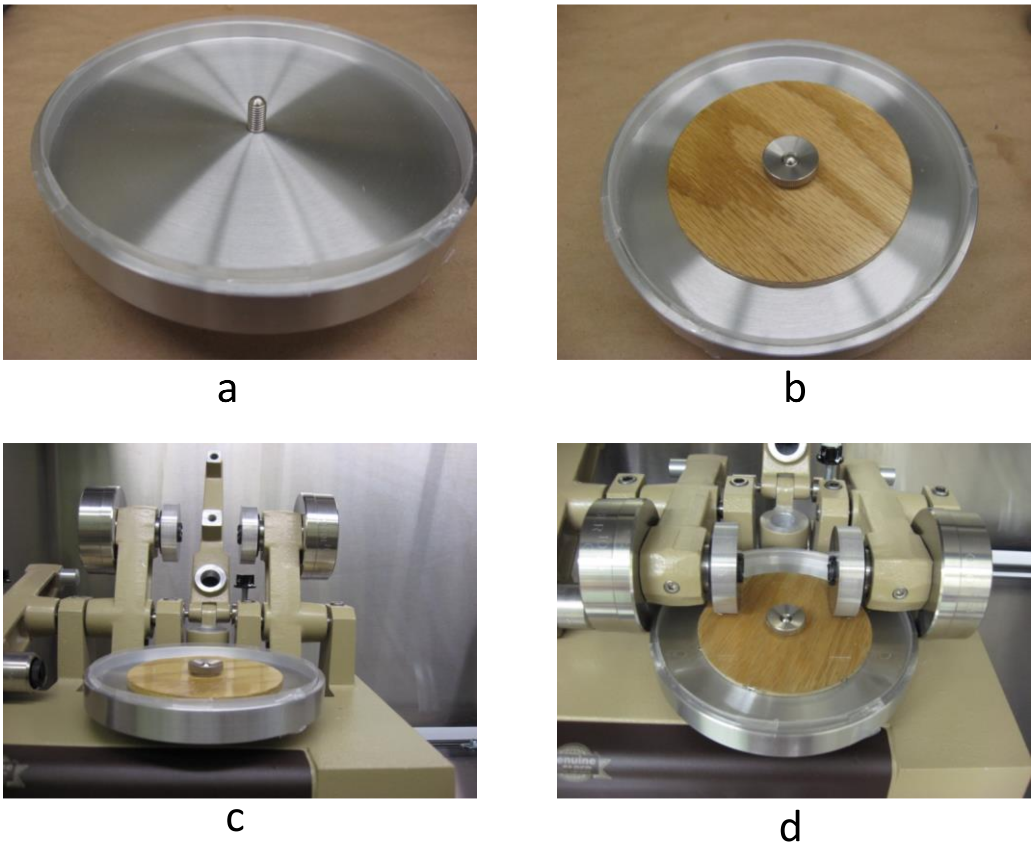

Figure S8. Instrument setup for abrasion in water:(**a**) accessory for abrasion in water; (**b**) a coated wood specimen (dia.= 9.6 cm) in the accessory; (**c**) a specimen on the accessory mounted on the rotary abraser, and (**d**) same as (**c**) but with addition of distilled water so that there was ≈ 1 mm of overlying water.

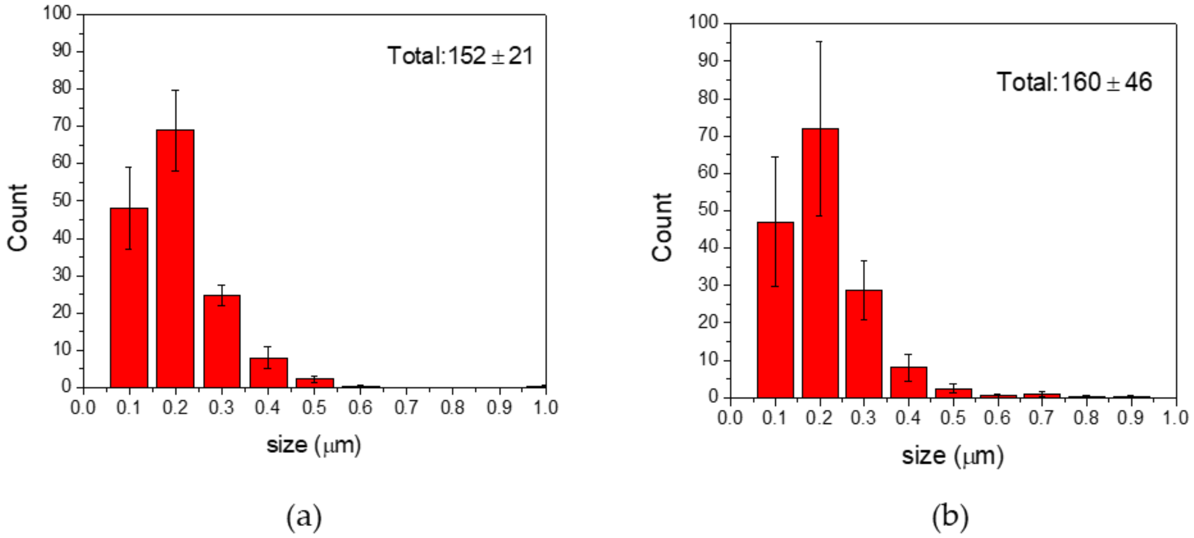

Figure S9. Effect of vacuum on the number and size distribution of surface-located released particles of the 1% nanoAl_2O_3 polyurethane floor coating; (**a**) with the vacuum on and (**b**) with no vacuum. A C1 wheel was used, and the abrasion parameters were the following: speed: 60 rpm, loading of 1000 g, number of cycles:100. Error bars represent one standard deviation. All measurements were conducted away from green particle cluster areas.

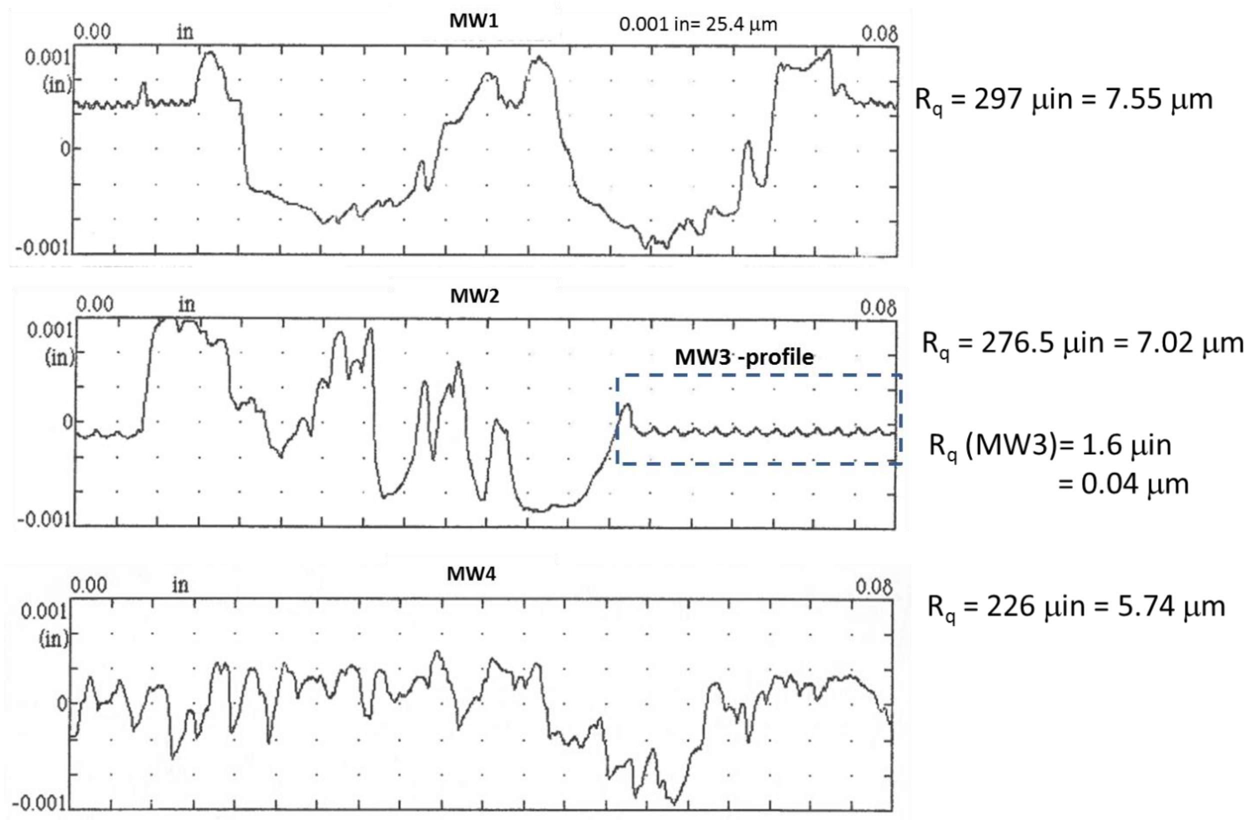

Figure S10. The surface profiles and roughness values of three different metallic wheels were measured with a profilometer: MW1, MW2 (including MW3), and MW4. Here R_q is the RMS (root-mean-square) roughness.

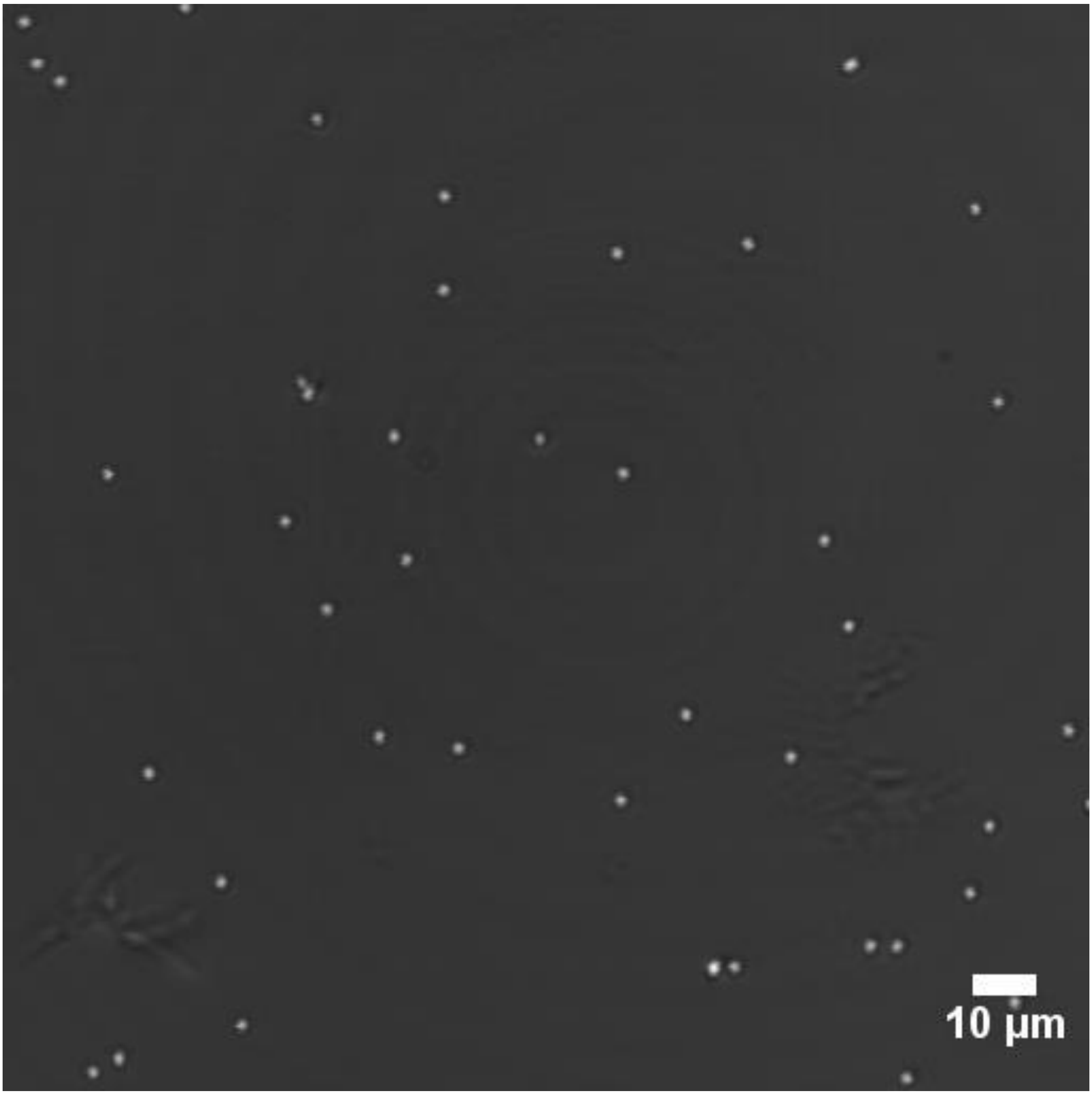

Figure S11. LSCM image at 50× (169 µm × 169 µm) of 300 nm diameter polystyrene beads. Despite being below the diffraction limit (308 nm) of the incident/reflected 543 nm light, these beads are detectable and their size can be estimated to be 300 nm. The size becomes more challenging to estimate with decreasing size but still can be estimated down to the smallest spot size of the detector, which is 80 nm.

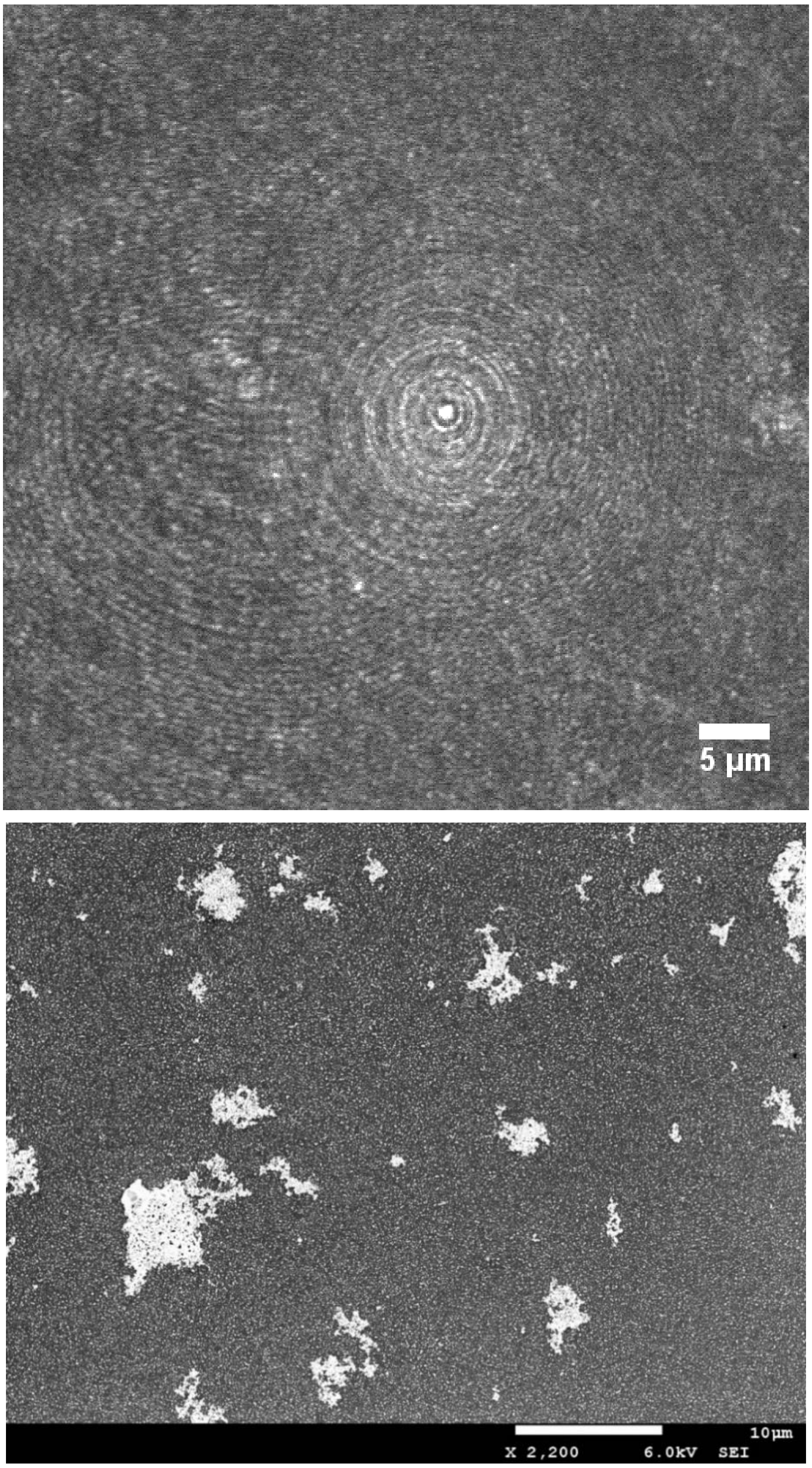

Figure S12. LSCM image (150×, 56 µm × 56 µm) (top) compared to SEM image (56 µm × 56 µm) of 50 nm particles (bottom), below the 80-nm spot size (or pixel size) possible with the LSCM detector. Particles with sizes below 80 nm can be observed with LSCM, but show up as bright spots that fill a pixel. With a lot of particles in close proximity to each other, neighboring pixels can give the illusion of larger particles being present.

© 2020 by the authors. Licensee MDPI, Basel, Switzerland. This article is an open access article distributed under the terms and conditions of the Creative Commons Attribution (CC BY) license (http://creativecommons.org/licenses/by/4.0/).
